# Heat shock transcription factor 1 protects against pressure overload-induced cardiac fibrosis via Smad3

**DOI:** 10.1007/s00109-016-1504-2

**Published:** 2017-01-13

**Authors:** Ning Zhou, Yong Ye, Xingxu Wang, Ben Ma, Jian Wu, Lei Li, Lin Wang, Dao Wen Wang, Yunzeng Zou

**Affiliations:** 1 Division of Cardiology, Department of Internal Medicine, Tongji Hospital, Tongji Medical College, Huazhong University of Science and Technology, 1095 Jiefang Avenue, Wuhan, 430030 China; 2 Shanghai Institute of Cardiovascular Diseases, Zhongshan Hospital and Institutes of Biological Science, Fudan University, Shanghai, 200032 China; 3 Division of Physiology, Department of Basic Sciences, School of Medicine, Loma Linda University, 11041 Campus Street, Loma Linda, 92354 CA USA

**Keywords:** Pressure overload, Cardiac fibrosis, Heat shock transcription factor 1, Smad3, Cardiac dysfunction, Fibroblasts

## Abstract

**Abstract:**

Fibrotic cardiac muscle exhibits high stiffness and low compliance which are major risk factors of heart failure. Although heat shock transcription factor 1 (HSF1) was identified as an intrinsic cardioprotective factor, the role that HSF1 plays in cardiac fibrosis remains unclear. Our study aims to investigate the role of HSF1 in pressure overload-induced cardiac fibrosis and the underlying mechanism. HSF1 phosphorylation was significantly downregulated in transverse aortic constriction (TAC)-treated mouse hearts and mechanically stretched cardiac fibroblasts (cFBs). HSF1 transgenic (TG) mice, HSF1 deficient heterozygote (KO) mice, and their wild-type littermates were subjected to sham or TAC surgery for 4 weeks. HSF1 overexpression significantly attenuated pressure overload-induced cardiac fibrosis and dysfunction. Conversely, HSF1 KO mice showed deteriorated fibrotic response and cardiac dysfunction upon TAC. Moreover, we uncovered that overexpression of HSF1 protected against fibrotic response of cFBs to pressure overload. Mechanistically, we observed that the phosphorylation and the nuclear distribution of the Smad family member 3 (Smad3) were significantly decreased in HSF1-overexpressing mouse hearts, while being greatly increased in HSF1 KO mouse hearts upon TAC, compared to the control hearts, respectively. Similar alteration of Smad3 phosphorylation and nuclear distribution were found in isolated mouse cardiac fibroblasts and mechanically stretched cFBs. Constitutively active Smad3 blocked the anti-fibrotic effect of HSF1 in cFBs. Furthermore, we found a direct binding of phosphorylated HSF1 and Smad3, which can be suppressed by mechanical stress. In conclusion, the present study demonstrated for the first time that HSF1 acts as a novel negative regulator of cardiac fibrosis by blocking Smad3 activation.

**Key messages:**

- HSF1 activity is decreased in fibrotic hearts.
- HSF1 overexpression attenuates pressure overload-induced cardiac fibrosis and dysfunction.
- Deficiency of HSF1 deteriorates fibrotic response and cardiac dysfunction upon TAC.
- HSF1 inhibits phosphorylation and nuclear distribution of Smad3 via direct binding to Smad3.
- Active Smad3 blocks the anti-fibrotic effect of HSF1.

**Electronic supplementary material:**

The online version of this article (doi:10.1007/s00109-016-1504-2) contains supplementary material, which is available to authorized users.

## Introduction

Cardiac dysfunction is the most common outcome of longstanding biophysical and biochemical stimuli. Despite recent inspiring advances, it remains a leading cause of death worldwide among elder adults [1]. Pressure overload is a detrimental biomechanical stress in primary hypertension and some valvular heart diseases, and provokes a broad array of molecular signaling cascades, leading to cardiac damages including myocardial hypertrophy, cardiomyocyte death, interstitial fibrosis, ventricular chamber enlargement, and eventually, heart failure [2, 3]. Accumulating data indicate that cardiac fibrosis is largely to be blamed for the pathophysiological changes of heart failure [4, 5]. As an important character of cardiac dysfunction, cardiac fibrosis aggravates cardiac stiffness and impairs cardiac compliance, eventually resulting in electromechanical coupling disorders and heart failure [4].

Smad family member 3 (Smad3) is considered as a central mediator of the myofibroblast transdifferentiation and extracellular matrix (ECM) synthesis in pressure overload-induced cardiac fibrosis [6]. In angiotensin II (AngII) and pressure overload-induced hypertensive mice, Smad3 deficiency significantly abrogated collagen deposition and inflammation [7, 8]. Multiple cytokines secreted by cardiomyocytes are involved in cardiac fibrosis and heart failure through modulating the phosphorylation of Smad3 [9]. Thus, Smad3 has been recently considered as a promising therapeutic target of cardiac fibrosis and consequent cardiac stiffness and dysfunction.

Heat shock transcription factor 1 (HSF1) is a transcription factor of the heat shock genes activated after temperature stress. HSF1 exists in cytoplasm as an inactive monomer in a complex with HSP40/HSP70 and HSP90. Phosphorylation of HSF1 on Ser230 by heat has been shown to contribute to the transcriptional activity of HSF1. Upon stress, HSF1 trimerizes after dissociating from the chaperone complex [10]. Activated HSF1 translocates into the nucleus and binds to the DNA containing heat shock elements (NGAAN) to activate its target genes [11]. It was demonstrated as a key factor involved in the adaptive mechanism of transition from adaptive cardiac hypertrophy to maladaptive heart failure [12], as well as an essential intrinsic factor protecting the cardiomyocytes against ischemic injury via interaction with signal transducer and activator of transcription 3 (STAT3) [10, 13]. HSF1 was found to be activated in the early phase of pressure overload and deactivated in the chronic phase (the maladaptive phase) [12]. We also showed that overexpression of HSF1 promotes cardiac angiogenesis and blunts cardiac dysfunction during chronic pressure overload [14]. Considering the general and complicated interactive network of HSF1 with other signaling molecules in pathological conditions [9, 15], it would be expected that there may exist an unrevealed association between HSF1 and Smad3 pathway.

In the present study, we used HSF1-overexpressing and HSF1-deficient mice in vivo, and isolated mouse cardiac fibroblasts (cFBs) with gain and loss of HSF1 function in vitro to investigate the role of HSF1 in cardiac fibrosis induced by pressure overload, and its underlying mechanisms. We showed for the first time that HSF1 acts as novel negative regulator of cardiac fibrosis by blocking Smad3 activation.

## Materials and methods

### Animal models

The generation of HSF1 - transgenic (TG) and HSF1-deficient heterozygote (KO) mice have been described previously [11, 16]. HSF1 TG and HSF1 KO mice and their respective wild-type littermates (WT) (aged from 8 to 10 weeks) were used in this study. For chronic pressure overload model, mice were subjected to TAC or sham operation as previously described [2, 15]. Briefly, mice were injected intraperitoneally with ketamine (25 mg/kg) and were administered 1.5% isoflurane intranasally for the maintenance of anesthesia during surgery. Mouse horizontal skin incision was made at the level of 2–3 intercostal space. The descending aorta was isolated, and a 6–0 silk suture was snared and pulled back around the aorta. A bent 27-gauge needle was then placed next to the aorta, and the suture was tied snugly around the needle and the aorta. After ligation, the needle was quickly removed, the chest and skin were closed, and the mice were allowed to recover. Sham-operated mice underwent the same procedure without constriction. Mouse hearts were excised at 4 weeks after TAC. All animal experimental protocols were approved by the Animal Care and Use Committee of Fudan University and in compliance with “Guide for the Care and Use of Laboratory Animals (the Guide, NRC 2011).”

### Culture and treatments of adult mouse cardiac fibroblasts

Mice were euthanatized by inhalation of 100% CO_2_ for 5 min. The mouse hearts were washed by ice-cold PBS to remove the blood. The mixture of heart fractions and digestion buffer were left for 1 min undisturbed. The first supernatant that contained debris and blood cells were discarded. The remaining deposits were digested with 25-ml digestion buffer under constant stirring at 37 °C for 10 min. Supernatant was collected into an icy tube containing fibroblast medium (10% FBS, 100 U/ml PenStrep, 1× L-glutamine, 100-μM ascorbic acid in DMEM/F12). Digestion was repeated until all the tissue has dissolved. The digested tissue mixtures were spun and re-suspended in fibroblast medium. The cell suspensions were pre-plated and cultivated for 2–3 days. The third or fourth passage of the fibroblasts was used in the present study. Mouse cFBs were cultured in silicon-based plates for 24 h and then mechanically stretched to 120% followed by treatment with adenovirus as described previously [15]. To overexpress Smad3, we used replication-defective adenoviral vectors encompassing the entire coding region of Smad3 gene under the control of the cytomegalovirus promoter. A similar adenoviral vector encoding the green fluorescent protein (GFP) gene was used as a control. We infected the cells with AdGFP or AdSmad3 at a multiplicity of infection (MOI) of 100, which resulted in transgene expression without toxicity in 95–100% of the cells. Mouse cFBs were mechanically stretched for 24 h after the adenovirus infection.

### Echocardiographic and hemodynamic measurements

Echocardiography and invasive hemodynamic measurement were performed at 2 or 4 weeks after sham or TAC surgery. Mice were anesthetized by inhalation of 2% isoflurane. Transthoracic echocardiographic analysis was performed by using an animal-specific instrument (VisualSonics Vevo770, VisualSonics Inc., Canada) as previously described [15]. Mice were anesthetized and M-mode images of the left ventricle (LV) were recorded. Cardiac structural and functional parameters included left ventricular internal end-diastolic dimensions (LVIDd), left ventricular anterior wall end-diastolic thickness (LVAWd), and left ventricular ejection fraction (LVEF). All measurements were averaged for five consecutive cardiac cycles and were carried out by three experienced technicians who were unaware of the identities of the respective experimental groups. Hemodynamic assessment was performed by a 1.4-F pressure catheter (SPR 671, Millar Instruments) inserted into the aorta and LV through the right common carotid artery after mice were anesthetized by inhalation of 2% isoflurane. The transducer was connected to Powerlab system (AD Instruments, Castle Hill, Australia) to record cardiac morphological and hemodynamic parameters.

### Histological analysis

Mice were euthanatized by using 100% CO_2_ inhalation for 5 min. For global morphometry, the hearts were perfused with PBS followed by 4% paraformaldehyde. For histological analysis, left ventricular tissues were fixed in 10% formalin and embedded in paraffin, sectioned at 5-μm thickness. The myocyte cross-sectional area (CSA) was measured with a quantitative digital image analysis system (Image-Pro Plus 6.0) using images that were captured from FITC-conjugated wheat germ agglutinin (WGA, Invitrogen Corp)-stained sections. More than 100 myocytes in the examined sections were outlined for each group of mice. Evidence of interstitial and perivascular collagen deposition was visualized by using picrosirius red (PSR) and Masson’s trichrome staining by light microscopy, respectively. Myocardial fibrosis area (MFA) and perivascular fibrosis area (PVF) were calculated by the following formula: MFA = the collagen area/ventricular wall area and PVF = perivascular collagen area/the vascular wall area, respectively. For measurements, five random high-power fields from each section were chosen and quantified in a blinded manner. Five sections for each heart were counted. The original images were measured by using an automated image analysis system (Image-Pro Plus5.0, Media Cybernetics, USA). All histological quantifications were performed by two observers in a blinded manner.

### Real-time quantitative RT-PCR

Total RNA was prepared from the left ventricular tissues of human hearts, mouse hearts, or cultured cardiac fibroblasts (cFBs) by using TRIzol reagent (Invitrogen, catalogue 15596–018, USA). cDNA was synthesized from 1-mg RNA of each sample by using the TOYOBO ReverTra Ace-α-RT-PCR kit according to the manufacturer’s instruction. Real-time PCR was performed on a Bio-Rad IQ5 multicolor detection system by using synthesized cDNA. A comparative CT method was used to determine relative quantification of special RNA levels. All real-time PCRs were performed at least in triplicates. Collagen type I (Coll. I), collagen type III (Coll. III), atrial natriuretic peptide (ANP), B-type natriuretic peptide (BNP), and glyceraldehyde-3-phosphate dehydrogenase (*GAPDH*) were amplified by using their specific primers listed in supplementary table 1.

### Co-immunoprecipitation and Western blot

Total protein of cultured cFBs and homogenized left ventricular tissues were extracted by the methods described previously [15]. Proteins of cellular nucleus were extracted using the Nuclear Extraction Kit (Millipore Inc., USA) according to the manufacturer’s instruction. The concentration of protein was measured by Bicinchoninic Acid Assay kit (Sigma-Aldrich, USA). A total 500-μg protein was used for co-immunoprecipitation. The immuno-complexes were precipitated with protein A/G Plus-Agarose (Santa Cruz Biotechnology Inc, USA), anti-total HSF1, and anti-total Smad3. The immuno-complexes were then subjected to the SDS-PAGE for detecting amounts of binding Smad3 and HSF1. To test the stability of the Co-IP system, the protein extracts of the WT mouse LV were used for the input. The samples of negative control were from protein extracts without the incubation of respective primary antibodies. The level of phosphorylated HSF1, total HSF1, phosphorylated Smad3, total Smad3, Coll. I, Coll. III, and Lamin B1 (LMNB1) were detected by Western blot (all primary antibodies were purchased from Santa Cruz Biotechnology Inc, USA). GAPDH detected by Western blot (Kangcheng Bio-Tech Inc. China) served as the internal control. The immunoreactivity was detected by Pro-Light chemiluminescent detection kit (Tiangen Biotech Inc, China) with LAS-3000 imaging system (Fujifilm Inc, Japan).

### Glutathione S-transferase (GST) pull-down assay

GST fusion HSF1, Smad3, and GST control proteins were expressed in *Escherichia coli* BL21 cells [17]. Briefly, following induction with 0.3 mM of isopropyl-b-D-thiogalactoside (IPTG) overnight at 16 °C, GST fusion proteins were purified from bacterial crude cell lysates according to the manufacturer’s instructions (Pierce, USA). Binding assays were performed by pre-incubating the GST or GST fusion HSF1 (Smad3) proteins beads with 100 μg/ml bovine serum albumin (BSA) in a binding buffer (50 mM Tris pH 7.5, 28 μM ZnCl_2_, 1% Triton ×100, 220 mM NaCl, 10% glycerol) at 4 °C for 1 h. The beads were centrifuged, re-suspended in binding buffer and incubated with 10 ng of recombinant Smad3 (HSF1) at 4 °C for 1 h. Beads were then washed three times with binding buffer containing 500 mM NaCl. The beads were mixed with 1× SDS-PAGE sample loading buffer and incubated at 95 °C for 10 min. The protein mixtures were separated on a 4 to 20% polyacrylamide gel. The protein bands were blotted onto a nitrocellulose membrane and probed using a Smad3 or HSF1 antibody.

### Statistical analysis

Data are shown as mean ± SEM. Multiple group comparison was performed by one-way or two-way ANOVA followed by LSD procedure for comparison of means. Comparison between two groups under identical conditions was performed by the two-tailed Student’s *t* test. A value of *P* < 0.05 was considered statistically significant.

## Results

### HSF1 activity was decreased in fibrotic hearts and cardiac fibroblasts after pressure overload

**Fig. 1:**
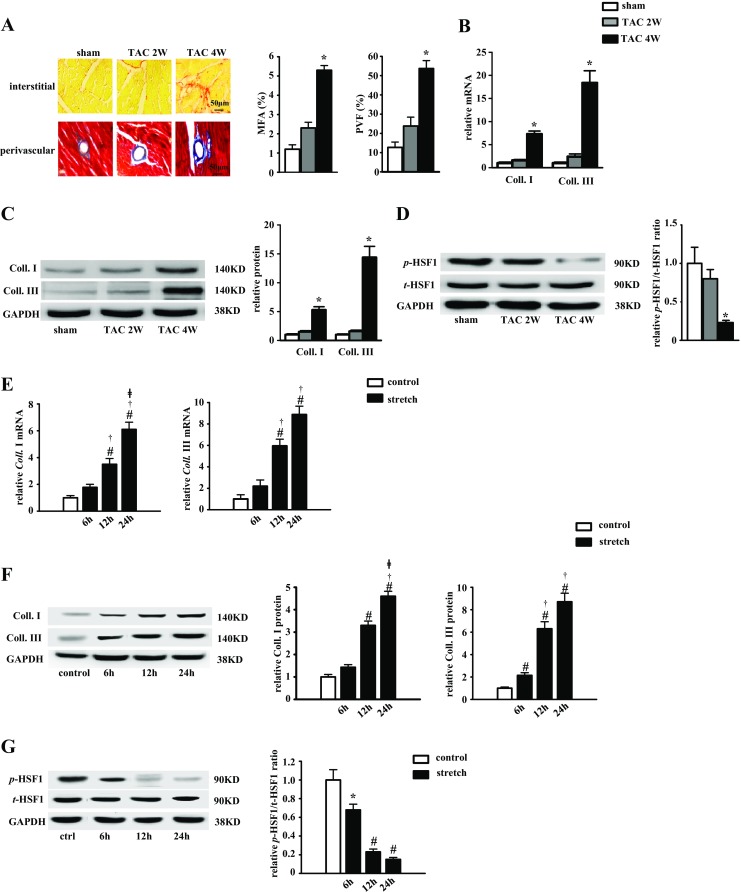
HSF1 phosphorylation was decreased in the fibrotic mouse hearts and mechanically stretched cFBs. **a** Histological sections of the left ventricles of C57BL/6 mice (*n* = 6) were stained with picrosirius red or Masson’s trichrome at 2 and 4 weeks after TAC surgery. *Left*, representative images; *right*, quantitative results. *Scale bar* 50 μm. Fibrotic areas of the histological sections were quantified using an image analysis system. *MFA* myocardial fibrosis area, *PVF* perivascular fibrosis area. **b** Real-time PCR analysis of Coll. I and Coll. III in C57BL/6 mouse hearts after sham or TAC surgery (*n* = 6). **c**, **d** Representative Western blots of Coll. I, Coll. III, total HSF1, and phosphorylated HSF1 in the mouse hearts after sham or TAC surgery (*n* = 6). **e** Real-time PCR analysis of Coll. I and Coll. III in 10-week-old C57BL/6 mouse heart cFBs after mechanical stretch for different durations. **f**, **g** Representative Western blots of Coll. I, Coll. III, total HSF1, and phosphorylated HSF1 in cFBs (*n* = 6). Values represent mean ± SEM. ^*^ *P* < 0.01 compared with sham mouse hearts. ^#^ *P* < 0.01 compared with control. ^†^ *P* < 0.01 compared with stretch for 6 h. ^ǂ^ *P* < 0.05 compared with stretch for 12 h. Two-way ANOVA test was used

To address whether HSF1 was involved in the myocardial fibrotic response to mechanical stress, we tested whether pressure overload inactivates HSF1 in vivo. C57BL/6 mice were subjected to TAC surgery. At 2 weeks after TAC, we observed significant concentric cardiac hypertrophy manifested with increased LV wall thickness, LV weight/tibia length (LVW/TL), and cross-sectional area (CSA), but preserved LV internal diameter end diastole (LVIDd) and LV ejection fraction (EF) (Supplementary Table 2). However, no remarkable fibrosis was observed in the TAC mouse hearts (Fig. 1a). Neither mRNA nor protein level of Coll. I and III were increased at 2 weeks after TAC (Fig. 1b, c). Western blot showed that the phosphorylation of HSF1 was preserved at 2 weeks after TAC (Fig. 1d).

Maladaptive cardiac hypertrophy and exacerbated cardiac dysfunction, evaluated by echocardiography and hemodynamic measurement, were found in mice at 4 weeks after TAC (Supplementary Table 2). Expectedly, significant cardiac interstitial and perivascular fibrosis was observed in mice at 4 weeks after TAC (Fig. 1a). Accordingly, the mRNA and protein level of Coll. I and Coll. III were all notably increased (Fig. 1b, c). With the development of the cardiac fibrosis, the phosphorylation of HSF1 was decreased (Fig. 1d).

Furthermore, in vitro-cultured adult WT mouse cFBs were subjected to mechanical stretch in the silicon plates as described previously [18]. The mRNA expressions of fibrotic markers including Coll. I and Coll. III were significantly increased by mechanical stretch time dependently (Fig. 1e). Accordingly, the protein levels of Coll. I and Coll. III were both increased by mechanical stretch (Fig. 1f). Inversely, the phosphorylation of HSF1 was significantly decreased by mechanical stretch in a time-dependent manner (Fig. 1g).

To explore the potential role of HSF1 in the human cardiac fibrosis, we analyzed the activation and expression of HSF1 in the fibrotic hearts from dilated cardiomyopathy (DCM) patients. Total proteins were extracted from LV samples of DCM patients undergoing heart transplantations from end-stage heart failure (HF) and those of donors. Increased mRNA levels of ANP, BNP, Coll. I, and Coll. III were found in the failing DCM hearts by using real-time PCR (Supplementary Fig. 1a). The protein levels of Coll. I and Coll. III were increased by 10.1-fold and 18.4-fold in failing hearts compared with donor hearts, respectively (Supplementary Fig. 1b). As Phosphorylation of HSF1 (*p*-HSF1) at Ser230 positively contributes to the transcriptional activity of HSF1 [19], the level of *p*-HSF1 at Ser230 was used to evaluate the activity of HSF1 in failing heart extracts. The level of *p*-HSF1 in the whole homogenate was reduced by 74% in human failing hearts compared to healthy hearts (Supplementary Fig. 1c). The phosphorylation of Smad3 was notably increased in fibrotic hearts (Supplementary Fig. 1d).

Taken together, the negative correlation between the HSF1 activation and fibrotic response to pressure overload revealed that HSF1 may be critically involved in the development of cardiac fibrosis.

### Overexpression of HSF1 inhibits pressure overload-induced cardiac fibrosis and consequently rescues cardiac dysfunction

The HSF1 TG mice overexpress a constitutively active form of HSF1. Although the level of total HSF1 are similar between the HSF1 TG mice and the WT littermates, Western blot analysis of the cardiac extracts revealed that the level of phosphorylation of HSF1 at Ser230 was increased by threefold in the HSF1 TG mice compared with the wild-type (WT) littermates (Supplementary Fig. 2).

**Fig. 2:**
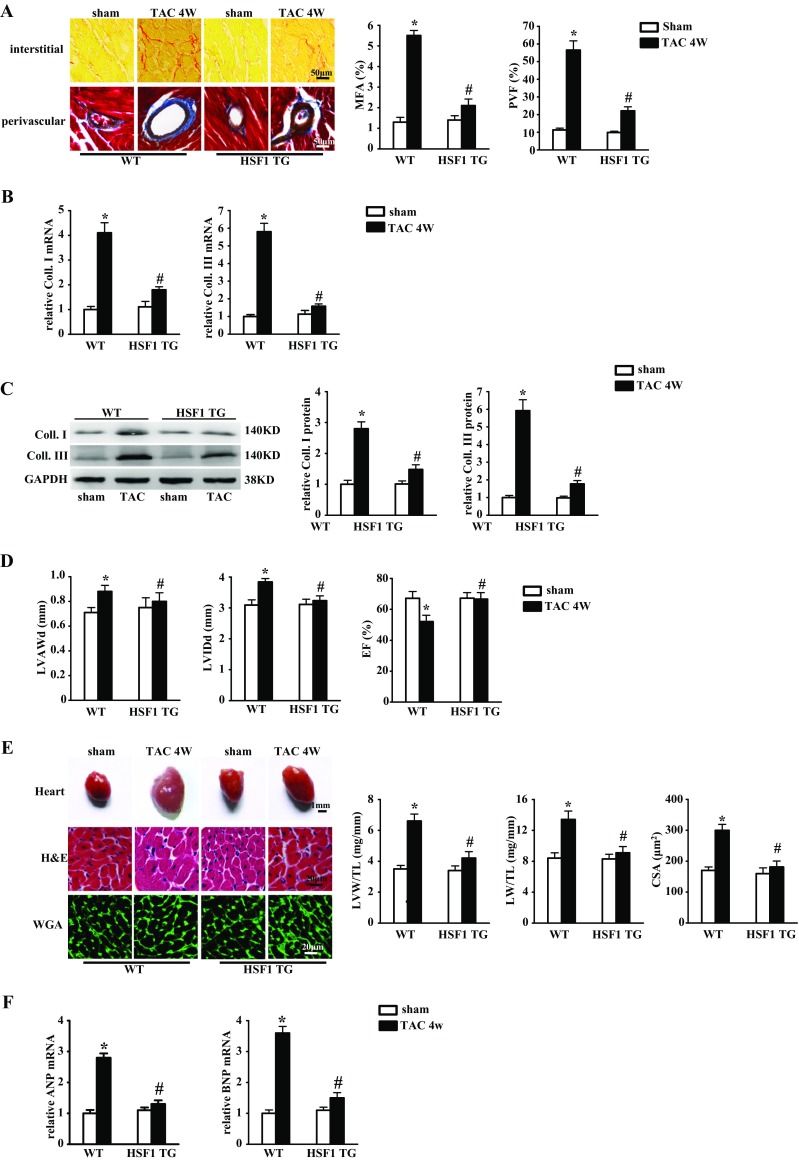
HSF1 TG mice were protected from TAC-induced cardiac fibrosis and cardiac dysfunction. **a** Picrosirius red and Masson’s trichrome staining of histological sections of the HSF1 TG and their WT littermate mouse left ventricles (LVs) at 4 weeks after TAC (*n* = 5 mice per experimental group; *scale bar* 50 μm). *Left*, representative images; *right*, quantitative results. Fibrotic areas of the histological sections were quantified using an image analysis system. *MFA* myocardial fibrosis area, *PVF* perivascular fibrosis area. **b** Real-time PCR analysis of Coll. I and Coll. III in mouse hearts after sham or TAC surgery (*n* = 5 per experimental group). **c** Representative Western blots of Coll. I, Coll. III, total HSF1, and phosphorylated HSF1 in indicated mouse LVs (*n* = 5 per experimental group). **d** Parameters of echocardiographic results for the WT and HSF1 TG mice. *LVAWd* left ventricular anterior wall end-diastolic thickness, *LVIDd* left ventricular internal end-diastolic dimensions, *LVEF* left ventricular ejection fraction (*n* = 5 per experimental group). **e** Histological analysis of whole hearts (*scale bar* = 1 mm), hematoxylin and eosin (HE)-stained (*scale bar* = 20 μm), and wheat germ agglutinin (WGA)-stained (*scale bar* = 20 μm) wild-type (WT) and HSF1 TG mouse hearts at 4 weeks after TAC surgery (*n* = 5 mice per experimental group; *scale bars* 20 μm). *Left*, representative images; *right*, quantitative results of the ratios of left ventricular weight/tibal length (LVW/TL), lung weight/tibal length (LW/TL), and myocardial cross-sectional area (CSA, *n* = 100+ cells per group). **f** Real-time PCR analysis of ANP and BNP induced by TAC in the indicated mice (*n* = 5 per experimental group). Values represent mean ± SEM. ^*^ *P* < 0.05 compared with WT sham; ^#^ *P* < 0.05 compared with WT TAC. Two-way ANOVA test was used

We found that HSF1 activity was decreased in pathological hearts, fibrotic mouse hearts, and mechanically-stretched cFBs; therefore, we decided to investigate the functional contribution of HSF1 to cardiac fibrosis in vivo. The HSF1 TG mice did not show any abnormalities in their cardiac morphology and functions at basal condition (Supplementary Table 3). WT and HSF1 TG mice showed similar increase of blood pressure at 4 weeks after TAC (Supplementary Table 4). However, significant cardiac interstitial and perivascular fibrosis were observed in the WT mouse hearts, which was notably reduced by 62 and 58% in the HSF1 TG mice, respectively (Fig. 2a). These results were confirmed by the mRNA levels of fibrotic markers, Coll. I and Coll. III (Fig. 2b). The protein levels of Coll. I and Coll. III were both significantly restrained in the HSF1 TG mice compared with their WT littermates (Fig. 2c), indicating an attenuated fibrotic response to pressure overload in the HSF1 TG mouse hearts compared with the WT mice.

At 4 weeks after TAC, the HSF1 TG mouse cardiac function was preserved compared with the WT mice evidenced by the measurement of echocardiography (Fig. 2d). Also, decreased LVW/TL ratio, LW/TL ratio, CSA, and downregulated mRNA expression of hypertrophic markers such as ANP and BNP were observed in the HSF1 TG mice compared with the WT mice, implicating an anti-hypertrophic effect of HSF1 against pressure overload (Fig. 2e, f). Taken together, these findings uncovered that HSF1 overexpression is capable of suppressing pressure overload-induced cardiac fibrosis and maladaptive cardiac hypertrophy.

### Deficiency of HSF1 exaggerates cardiac fibrosis and dysfunction induced by pressure overload

Given the evidence indicating that HSF1 overexpression inhibits cardiac fibrosis upon pressure overload, we sought to determine whether a deficiency of HSF1 could augment pressure overload-induced cardiac fibrosis. To this end, the HSF1 KO mice were used in this study. The HSF1 KO mice did not show any abnormalities in their cardiac morphology and functions (Supplementary Table 5). The level of phosphorylation of HSF1 in cardiac is reduced by a striking 71% in the HSF1 KO mice compared with the WT littermates despite of a preserved protein level of total HSF1 (Supplementary Fig. 2).

**Fig. 3:**
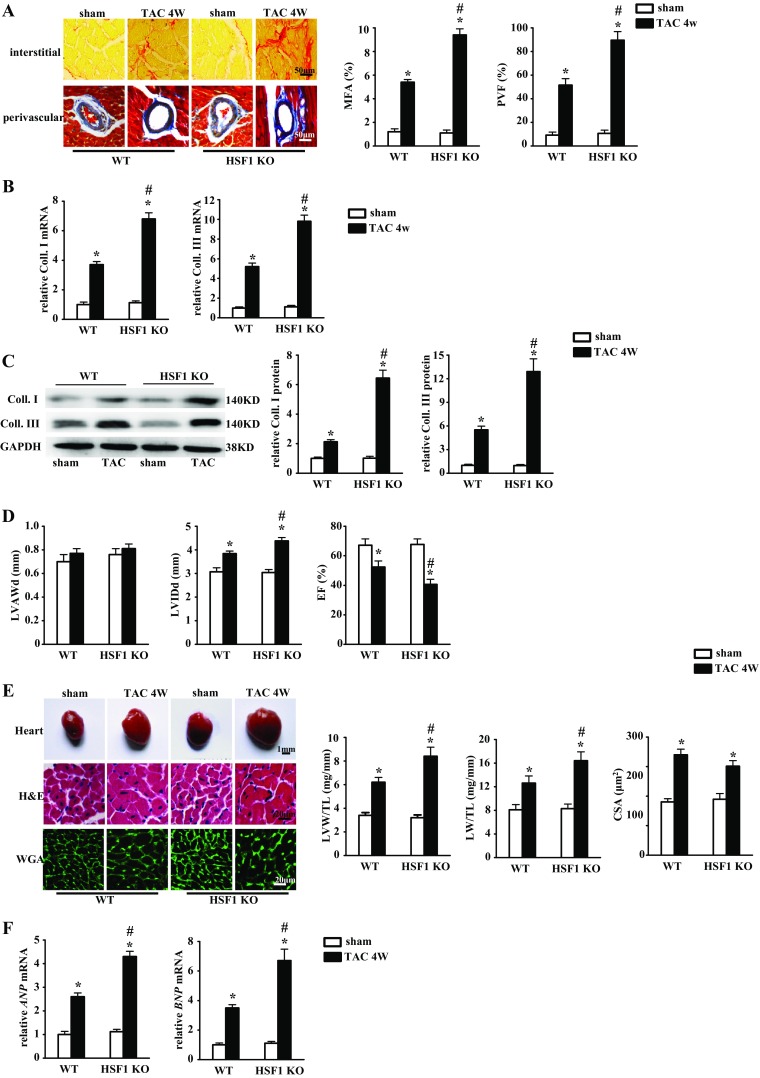
Deficiency of HSF1 exaggerated pressure overload-induced cardiac fibrosis. (**a**) Picrosirius red and Masson’s trichrome staining of histological sections of the HSF1 TG and their WT littermate mouse left ventricles (LVs) at 4 weeks after TAC (*n* = 5 mice per experimental group; *scale bar* 50 μm). *Left*, representative images; *right*, quantitative results. Fibrotic areas of the histological sections were quantified using an image analysis system. *MFA* myocardial fibrosis area, *PVF* perivascular fibrosis area. **b** Real-time PCR analysis of Coll. I and Coll. III in mouse hearts after sham or TAC surgery (*n* = 5 per experimental group). **c** Representative Western blots of Coll. I, Coll. III, total HSF1, and phosphorylated HSF1 in mouse hearts (*n* = 5 per experimental group). **d** Parameters of echocardiographic results of the WT and the HSF1 KO mice. *LVAWd* left ventricular anterior wall end-diastolic thickness, *LVIDd* left ventricular internal end-diastolic dimensions, *LVEF* left ventricular ejection fraction (*n* = 5 per experimental group). **e** Histological analysis of whole hearts (*scale bar* = 2 mm), hematoxylin and eosin (HE)-stained (*scale bar* = 20 μm), and wheat germ agglutinin (WGA)-stained (*scale bar* = 20 μm) wild-type (WT) and HSF1 KO mouse hearts at 4 weeks after TAC surgery (*n* = 5 mice per experimental group; *scale bars* 20 μm). *Left*, representative images; *right*, quantitative results of the ratios of left ventricular weight/tibal length (LVW/TL), lung weight/tibal length (LW/TL), and myocardial cross-sectional area (CSA, *n* = 100+ cells per group). **f** Real-time PCR analysis of hypertrophic markers, ANP, and BNP induced by TAC in the indicated mice (*n* = 5 per experimental group). Values represent mean ± SEM. ^*^ *P* < 0.05 compared with respective sham; ^#^ *P* < 0.05 compared with WT TAC. Two-way ANOVA test was used

Heart rate and body weights were preserved in the WT and the HSF1 KO mice although the blood pressure was significantly increased after TAC for 4 weeks (Supplementary Table 6). At 4 weeks after TAC, both MFA and PVF in the hearts of the HSF1 KO mice were increased by 1.7-fold compared with the hearts from the WT littermates, indicating an aggravated cardiac fibrosis in the hearts of the HSF1 KO mice (Fig. 3a). Consistent with the above histological test, the expressions of fibrotic markers Coll. I and Coll. III at both mRNA and protein levels were significantly increased in the HSF1 KO mouse hearts (Fig. 3b, c). The HSF1 KO mice also displayed a pronounced eccentric cardiac hypertrophy manifested by notably increased LVIDd, decreased LVEF, and preserved LVAWd compared with the WT mice (Fig. 3d). Accordingly, increased LVW/TL ratio, LW/TL ratio, and CSA as well as mRNA expression of ANP and BNP were observed in the HSF1 KO mice compared with the WT mice, suggesting an exaggerated maladaptive cardiac remodeling in the HSF1 - deficient mice treated by chronic pressure overload (Fig. 3e, f). These data together demonstrate that HSF1 deficiency promotes cardiac fibrosis and exacerbates the cardiac dysfunction in response to pressure overload.

We next tested the cardioprotective effect of HSF1 in another isoproterenol-induced cardiac fibrosis model. The HSF1 TG, HSF1 KO, and their WT littermates received continuous administration of isoproterenol or 0.9% saline for 4 weeks through subcutaneously implanted osmotic minipumps. Similar to the model of pressure overload-induced cardiac fibrosis, isoproterenol perfusion significantly increased MFA and PVF, as well as the mRNA expression of collagens I and III. These fibrotic responses were notably restrained in the HSF1 TG mice, while exaggerated in the HSF1 KO mice (Supplementary Tables 7 and 8).

### HSF1 inhibits mechanical stretch-induced fibrotic response in cardiac fibroblast

**Fig. 4:**
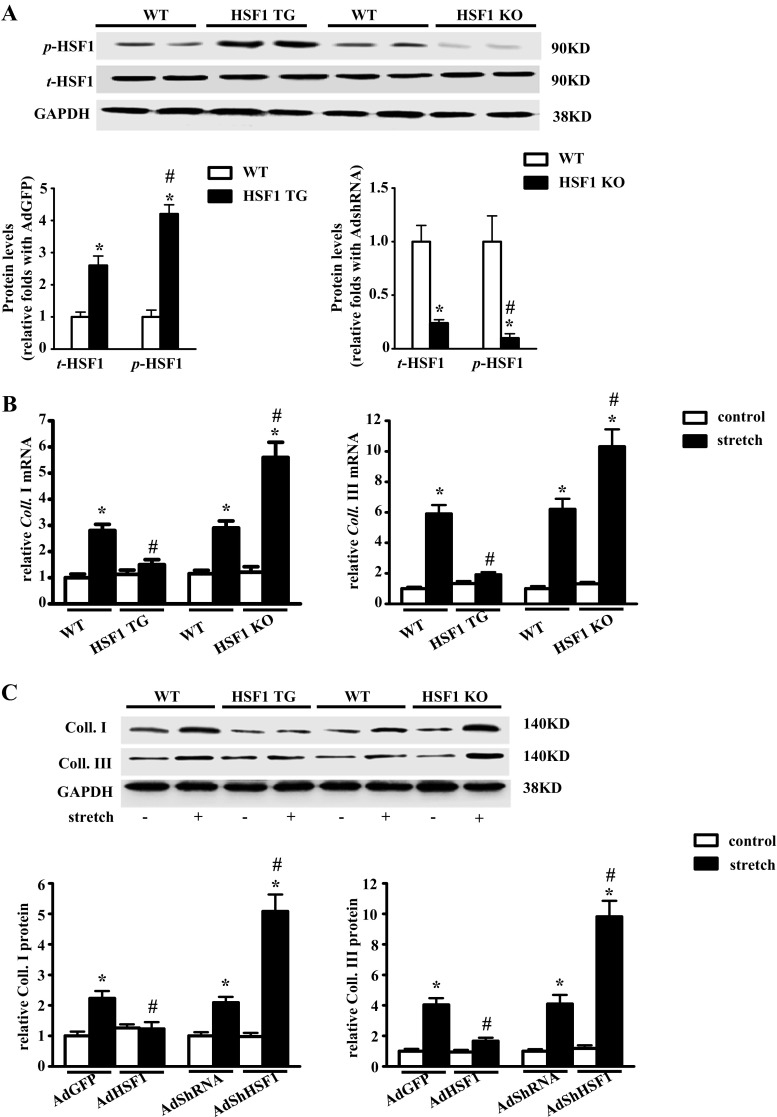
HSF1 inhibits mechanical stretch-induced fibrotic response in mouse cardiac fibroblast. **a** Representative Western blots of total and phosphorylated HSF1in cardiac fibroblasts from mouse with different genotypes (*n* = 4 independent experiments). *Up*, representative blots. *Below*, quantitative results. **b** Real-time PCR analysis of fibrotic markers (Coll. I and Coll. III) in cFBs from the HSF1 TG and KO mouse hearts after the treatment with mechanical stretch for 24 h (*n* = 4 independent experiments). **c** Representative Western blots and their quantitative results of Coll. I and Coll. III in cFBs (*n* = 4 independent experiments). Values represent mean ± SEM. ^*^ *P* < 0.05 vs. respective control; ^#^ *P* < 0.05 vs. respective stretch. Two-way ANOVA test was used

Considering that chronic overexpression or deletion of HSF1 in vivo may have compensatory effects, which influence the activity of HSF1 in pressure overload-induced cardiac fibrosis, we then performed ex vivo studies using cardiac fibroblasts isolated from WT, HSF1 TG, and HSF1 KO mouse hearts. We firstly measured the phosphorylation of HSF1 in the HSF1 TG, KO and their WT littermates. The phosphorylation level of HSF1 in the cardiac fibroblasts is increased by 4.2-fold in the HSF1 TG mouse heart, while decreased by 90% in the HSF1 KO mice. Mechanical stretch significantly increased both mRNA and protein levels of Coll. I and Coll. III. These fibrotic markers were both notably reduced in cFBs from the HSF1 TG mice, while boosted in cFBs from the HSF1 KO mice (Fig. 4b, c). Taken together, these findings indicate that HSF1 inhibits the fibrotic response in cFBs led by mechanical stress.

Considering the vital role of cardiomyocytes (CMs) in cardiac fibrosis [20], we also tested the effect of HSF1 on the fibrotic responses of CMs to mechanical stress. To address this issue, we first performed gain- and loss-of-function studies in cultured NRCMs. We infected NRCMs with either HSF1 adenovirus (AdHSF1) to overexpress HSF1 and increase HSF1 activity or HSF1 shRNA adenovirus (AdshHSF1) to knockdown HSF1 and decrease its activity. Sustained mechanical stretch for 24 h raised the level of both mRNA and protein of CTGF in NRCMs, which was markedly abrogated by infection of AdHSF1, whereas promoted by infection of AdshHSF1 (Supplementary Fig. 3).

### HSF1 inhibits the activation and nuclear translocation of Smad3

**Fig. 5:**
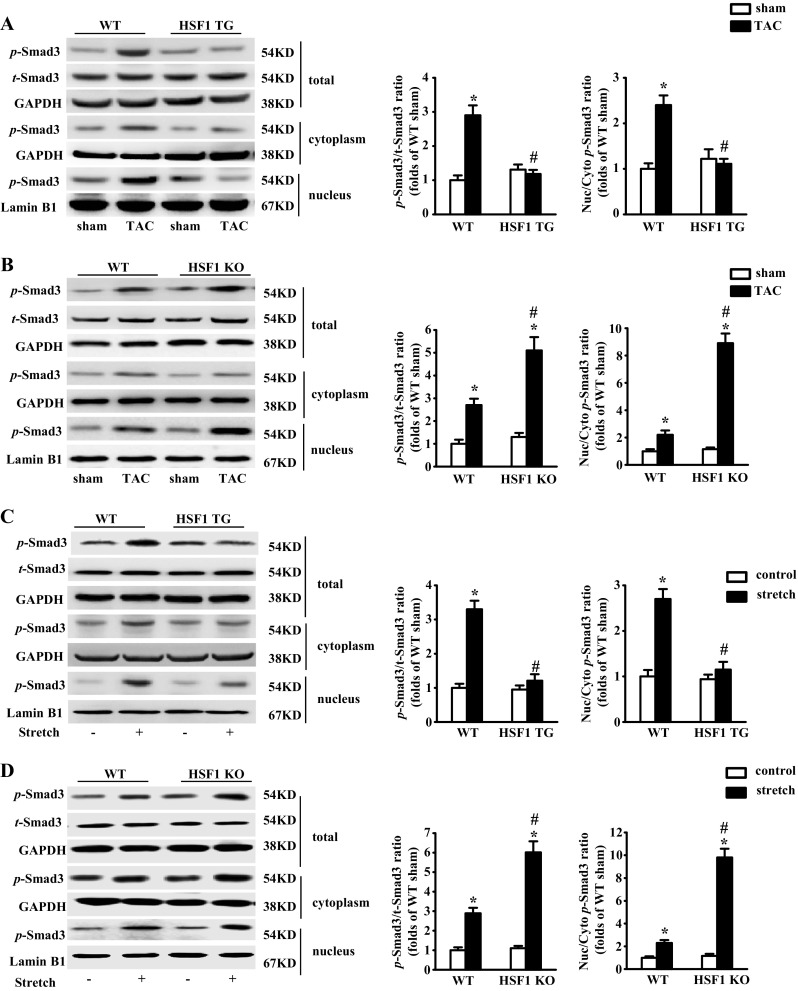
HSF1 inhibits the activation and nuclear translocation of Smad3. **a**, **b** Representative Western blots and quantitative results of the Smad3 phosphorylation and total levels in the HSF1 TG and KO mice 4 weeks after TAC, respectively (*n* = 5). **c**, **d** Representative Western blots and quantitative results of the Smad3 phosphorylation and total levels in the mechanically - stretched cFBs from the HSF1 TG and KO mice, respectively (*n* = 4 independent experiments). Values represent mean ± SEM.^*^ *P* < 0.05 vs. respective sham or control cFBs; ^#^ *P* < 0.05 vs. respective TAC or stretched cFBs. Two-way ANOVA test was used

Since Smad3 is a key transcriptional factor in cardiac fibrosis, we measured the phosphorylation of Smad3 in both the pressure overloaded mouse hearts and the mechanically stretched cFBs. We observed that 4 weeks after TAC, the phosphorylation of Smad3 was decreased by 59% in the HSF1 TG mouse hearts (Fig. 5a), whereas increased by 2.2-fold in the HSF1 KO mice compared with those hearts of the WT mice (Fig. 5b). Similarly, the phosphorylation of Smad3 in mechanical-stretched cFBs was reduced by 63% in HSF1 TG mice, while increased by 2.1-fold in the HSF1 KO mice, compared with the control cFBs (Fig. 5c, d).

Smad3 was phosphorylated at the SSXS motif in the C-terminal tail. Phosphorylated Smad3 translocated from cytoplasm into nucleus to modulate the development of fibrosis [21, 22]. The cytoplasmic and nuclear fraction of the mouse hearts and cultured neonatal rat cFBs were subjected to Western blot analysis in the present study. The phosphorylated levels of Smad3 in total protein both cytoplasmic extracts and nuclear fractions of the HSF1 TG mouse heart extracts were decreased compared with the WT mice (Fig. 5a). However, the decrease of Smad3 phosphorylation is more significant in nucleus than cytoplasm evidenced by 50% reduction of nucleus/cytoplasm concentration ratio of phosphorylated Smad3 in the HSF1 TG mice compared with the WT mice (Fig. 5a). Inversely, the nucleus/cytoplasm concentration ratio of phosphorylated Smad3 in the HSF1 KO mouse hearts increased by 3.2-fold compared with the WT mouse hearts, suggesting that the increased level of Smad3 phosphorylation in the HSF1 KO mouse heart was largely attributed to the increased phosphorylated Smad3 in nucleus (Fig. 5b). Similar to in vivo observations, the decrease of phosphorylated Smad3 in nucleus was more significant than the decrease in cytoplasm from mechanically stretched cFBs from the HSF1 TG mice (Fig. 5c), whereas HSF1 KO significantly increased the phosphorylated Smad3 in nucleus of cFBs imposed with mechanical stretch (Fig. 5c). These results together imply that HSF1 activity regulates Smad3 activation and nuclear translocation.

### HSF1-mediated anti-fibrotic effect is Smad3 dependent

**Fig. 6:**
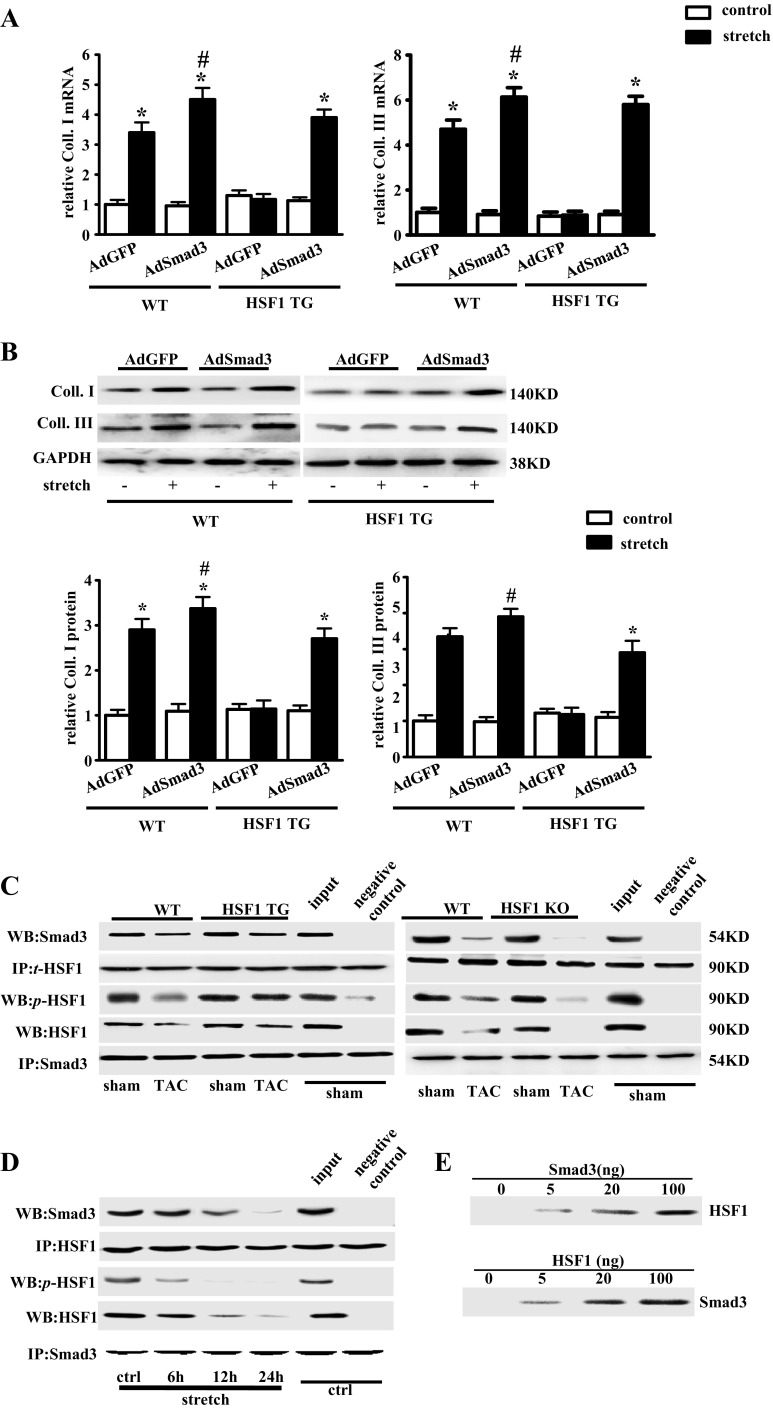
HSF1-mediated anti-fibrotic effect is Smad3 dependent. **a** Real-time PCR analysis of fibrotic markers (Coll. I and Coll. III) in cFBs transduced with indicated adenovirus and treated with mechanical stretch for 24 h (*n* = 4 independent experiments). **b** Representative Western blots and quantitative results of the fibrotic markers (Coll. I and Coll. III) in cFBs after transduction with indicated adenovirus and treatment with mechanical stretch for 24 h (*n* = 5 independent experiments). **c** Total proteins extracted from LV of indicated mice were immunoprecipitated (IP) with antibodies against *t*-HSF1 (*top*) or Smad3 (*bottom*) and then the immune complexes were subjected to Western blot (WB) analysis for Smad3 or HSF1, respectively. **d** Total protein was extracted from cultured cFBs from the WT mouse heart infected by indicated adenoviruses and mechanically- stretched for 6, 12, and 24 h, respectively. Total protein was use to do immunoprecipitation by using antibodies against HSF1 (*top*) or Smad3 (*bottom*), and then, the immune complexes were detected by Smad3 or HSF1 antibodies, respectively. **e** Glutathione S-transferase (GST) pull-down assay to detect the direct interaction between HSF1 and Smad3. Pull-down assay was performed with various concentrations of GST-HSF1 (0–100 ng) protein (*upper panel*) or GST-Smad3 (0–100 ng) protein (*lower panel*). Concentration-dependent increase in interaction between Smad3 and HSF1 protein was detected. Glutathione S-transferase (GST) was used as a loading control. Values represent mean ± SEM. ^*^ *P* < 0.05 vs. respective control; ^#^ *P* < 0.05 vs. respective stretch. Two-way ANOVA test was used

Next, we examined whether activation or inactivation of the Smad3 signaling cascade would affect the regulatory role of HSF1 in the development of cardiac fibrosis. To address this issue, the cFBs isolated from adult mouse hearts were infected with Smad3 adenovirus (AdSmad3) before mechanical stretch for 24 h. Both mRNA and protein levels of collagens I and III in cFBs from the WT mouse were significantly increased, which was promoted by infection of AdSmad3. Mechanical stretch did not increase the expression of these fibrotic markers in the HSF1 TG mouse heart cFBs infected with AdGFP. However, after infection of AdSmad3, these markers were significantly upregulated by mechanical stretch, indicating that inhibition of Smad3 is the key mechanism mediating the HSF1-induced anti-fibrotic effect (Fig. 6a, b).

As stress-induced Smad3 activation and nuclear translocation play critical roles in cardiac fibrosis and remodeling [8], our finding of HSF1-mediated Smad3 inhibition prompted us to examine whether HSF1 directly interacted with Smad3. We firstly performed co-immunoprecipitation (co-IP) experiments to test the binding of HSF1 and Smad3 in both the HSF1 TG and the HSF1 KO mice after TAC surgery. Expectedly, boosted bindings of HSF1 and Smad3 were detected in HSF1 TG mouse hearts, which was preserved after TAC for 4 weeks (Fig. 6c). However, much less binding was observed in HSF1 KO mouse hearts (Fig. 6c). Then we performed co-IP assay in cFBs treated by mechanical stretch for 24 h. We found that HSF1 could co-precipitate along with Smad3 which is significantly inhibited by mechanical stretch time dependently (Fig. 6d). GST pull-down assay showed that Smad3 and HSF1 bind directly (Fig. 6e). As HSP90/HSP70 physiologically binds with HSF1, we assumed that HSP90 and HSP70 also bind to Smad3. However, our results did not show any binding between HSP90/HSP70 and Smad3 (Supplementary Fig. 4). Taken together, these data suggested that Smad3 was regulated by HSF1 via direct interaction with HSF1.

## Discussion

In the present study, we for the first time identified HSF1 as an important protective regulator of cardiac fibrosis induced by pressure overload. We firstly unraveled that mechanical stress decreased the HSF1 activation both in vivo and in vitro in a time-dependent manner. We also found that HSF1 is inactivated in failing and fibrotic human hearts. In animal experiments, HSF1 TG mice showed alleviative cardiac fibrosis in response to pressure overload along with relived pathological cardiac hypertrophy and preserved cardiac function. In contrast, HSF1 KO mice exhibited deteriorated fibrotic responses and worsened cardiac dysfunction after TAC surgery. In ex vivo experiments, we demonstrated that HSF1 overexpression significantly inhibited mechanical stretch-induced fibrosis, whereas HSF1 knockdown increased this fibrotic response to mechanical stretch in cardiac fibroblasts. Overexpression of Smad3 blocked the anti-fibrotic effect of HSF1 in mechanical-stretched cardiac fibroblasts. We further illuminated that HSF1 binds directly to Smad3, thus regulating the activation and translocation of Smad3, which consequently inhibited cardiac fibrosis. Therefore, HSF1 upregulation may provide a new therapeutic strategy for the treatment of cardiac fibrosis induced by diseases such as hypertension, aortic valvular stenosis, and aortic constriction.

The mechanisms of cardiac fibrotic response to pressure overload are extremely complex, including cell membrane receptors, signal transduction pathways, and inflammation, as well as transcriptional and posttranscriptional regulation of genes [3]. Since cardiac fibrosis plays a critical role in the adverse outcomes of pathological cardiac remodeling, great efforts were exerted to look for cardioprotective cytokines to relieve cardiac fibrosis. HSF1, a serine-rich constitutively phosphorylated mediator of the stress response, is an important transcription factor for heat shock proteins and a definite protective factor in the heart [10, 23–25]. Upon stress, HSF1 forms trimers, relocalizes to nuclear granules, undergoes inducible phosphorylation, and acquires the properties of a transactivator. Ser230 is identified as a site of endogenous phosphorylation on human HSF1 located in the regulatory domain which promotes the magnitude of the inducible transcriptional activity of HSF1 [26].

So far, it is unknown whether HSF1 is involved in human cardiac fibrosis. One of our observations in the present study is that HSF1 activity was significantly reduced in fibrotic hearts from DCM patients compared with healthy donors. Cardiac fibrosis accounts, at least in part, for maladaptive cardiac remodeling in DCM patients [27]. Although pressure overload is not the cause of cardiac fibrosis of the DCM patients, we identified a negative relationship between cardiac fibrosis and the activity of HSF1.

In the present study, HSF1 phosphorylation was gradually decreased in the pressure overload-induced cardiac fibrosis time dependently in vivo and in vitro. The phosphorylation level of HSF1 in fibroblasts was significantly decreased within 12 h by mechanical stretch, while pressure overload could not change the phosphorylation level of HSF1 in ventricular tissues at 2 weeks. The following reasons may be responsible for the different response time to the mechanical stimuli between in vivo and in vitro studies. First of all, in the in vitro study, what we detected is the phosphorylation level of HSF1 in mechanical-stretched fibroblasts; however, the phosphorylation level of HSF1 detected in ventricular tissues was not only due to fibroblasts but also due to many other cells. Secondly, the adaptive neuroendocrine changes after TAC operation may attribute to the longer response of hearts to pressure overload. We thus concluded that pressure overload can restrain HSF1 activation.

Overexpression of HSF1 inhibits pressure overload-induced cardiac fibrosis and cardiac dysfunction. Conversely, the deficiency of HSF1 exaggerates pressure overload-induced cardiac fibrosis and dysfunction. Loss of HSF1 in the HSF1 KO mouse cardiac fibroblasts augmented fibrotic response of cFBs to mechanical stretch, indicating that the reduction of HSF1 activation may promote the progression of cardiac fibrosis. Meanwhile, our results also uncovered that increased phosphorylation level of HSF1 significantly abrogated the fibrotic response to mechanical stretch in cFBs. These findings together indicate that HSF1 activation is critically involved in the cardiac fibrotic response to pressure overload. Xie et al. showed that isoproterenol induced significant and similar cardiac fibrosis in the Kunming mice and the HSF1^−/+^ mice, which was very slight in the HSF1^−/−^ mice [28], indicating a detrimental effect of HSF1 on cardiac fibrosis induced by isoproterenol. However, we drew a different conclusion based on an amelioration of cardiac fibrosis in HSF1 TG mice treated with isoproterenol. In the past 10 years, other group’s studies together with ours have identified HSF1 as an endogenous cardioprotective factor [12–14, 23, 24, 29]. Actually, we had reported the protective effect of HSF1 against cardiac fibrosis induced by pressure overload in 2011 [14]; however, the mechanism is still unclear. Since our results are inconsistent with theirs, the following reasons should be considered. Different methods were used to evaluate the cardiac fibrosis. They used hematoxylin and eosin staining to observe fibrosis in the hearts. However, we used picrosirius red and Masson’s trichrome staining, which are two kinds of canonical methods evaluating organ fibrosis [30]. Furthermore, real-time PCR and WB detecting the collagens I and III were used to evaluate the cardiac fibrosis in the present study. The different observation methods may contribute to the different conclusions.

HSF1 plays a protective role in cardiac fibrosis induced by pressure overload. However, the underlying mechanism by which HSF1 prevents cardiac fibrosis and inhibits heart failure remains unclear. Accumulating evidences have indicated that numerous transcription factors including signal transducer and activator of transcription 3 (STAT3), protein kinase C (PKC), Smad3, Smad family member 4 (Smad4), and P38 are involved in the initiation and development of cardiac fibrosis [21, 31–34]. Therefore, it is worthy to examine whether the expressions/activities of these transcription factors are interfered by HSF1 in hearts upon chronic pressure overload. We detected the expression of STAT3, PKC, Smad3, Smad4, and P38 in the TAC-induced fibrotic hearts collected from both the HSF1TG and KO mice by immunoblotting (data not shown). Smad3 was the only one to be regulated in fibrotic hearts by HSF1. Thus, HSF1-mediated regulation of cardiac fibrosis may be directly associated with Smad3. As an essential downstream transcription factor of tissue growth factor β (TGFβ), Smad3 mediates TGFβ signaling to regulate cardiac fibrotic response to pressure overload and ischemic stimulations [6, 8, 35, 36]. Pressure overload, partly mediated by the angiotensin II receptor 1, activates the TGFβ receptor 1 and phosphorylates downstream Smad3 [8, 37]. After phosphorylation, Smad3 translocates from the cytoplasm to the nucleus and promotes the transcription of multiple pro-fibrotic factors including CTGF, periostin, and TGFβ [8, 21, 38]. Inhibition of Smad3 phosphorylation ameliorated the pressure overload-induced cardiac fibrosis and cardiac dysfunction in mouse [21]. Notably, under basal condition, either overexpression or inhibition of HSF1 in mouse hearts did not alter both the total and phosphorylated Smad3; however, on pressure overload, phosphorylated Smad3 was increased in the HSF1 KO mice, but decreased in the HSF1 TG mice. These data suggest that HSF1-mediated regulation of Smad3 might be stress dependent. Indeed, this is supported by our data showing that blockage of Smad3 activation offsets the HSF1-elicited fibrotic response. Currently, it is well documented that increased Smad3 expression results in the activation of pro-fibrotic gene programs (i.e., CTGF, SMA, and collagen), leading to cardiac fibrosis [5]. In addition, previous studies also suggested that the activation of Smad3 was essential for pressure overload or AngII-induced myocardial fibrosis [8, 20, 21, 39]. Together, the data from co-IP, GST pull-down and immunostaining assays together indicated an interaction between *p*-HSF1 and unphoshoryated Smad3. Although our results might not exclude other mechanisms by which HSF1 suppresses cardiac fibrosis induced by pressure overload, the inhibitory effect of HSF1 on the development of cardiac fibrosis seems to be largely dependent on the suppression of Smad3 by HSF1.

**Fig. 7:**
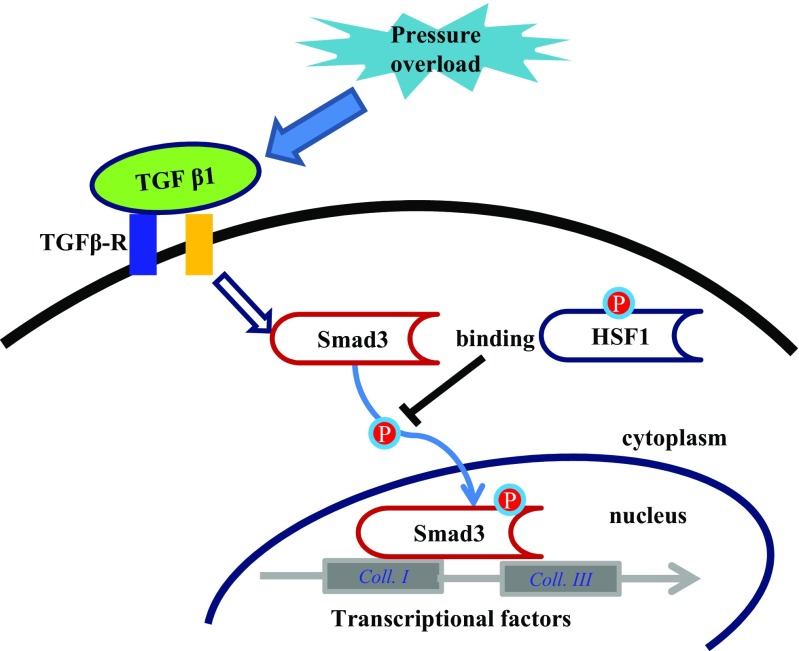
A diagram describing HSF1 mediated amelioration of cardiac fibrosis

In conclusion, our present work provides the first evidence that cardiac HSF1 activation inhibits cardiac fibrosis in response to pressure overload via inhibition of the Smad3 phosphorylation and translocation (Fig. 7). We propose that targeting of the HSF1 may develop novel promising strategies for the treatment of cardiac fibrosis and dysfunction in patients with disease such as hypertension, aortic valvular stenosis, and aortic constriction.

## Electronic supplementary material

Below is the link to the electronic supplementary material.

ESM 1 (DOC 1.15 mb)
