# Supplementary material for: Heat shock transcription factor 1 protects against pressure overload-induced cardiac fibrosis via Smad3
Source: J Mol Med (Berl). 2017 Jan 13;95(4):445–60. doi: 10.1007/s00109-016-1504-2 (PMC5357304; doi:10.1007/s00109-016-1504-2)
Supplement: Supplementary file 1 — (DOC 1.15 mb) [file 109_2016_1504_MOESM1_ESM.doc]

**Supplementary materials**

**Heat shock transcription factor 1** **protects against pressure overload induced-cardiac fibrosis via Smad3**

Ning Zhou ^1^, Yong Ye ^2^, Xingxu Wang ^2^, Ben Ma^3^, Jian Wu ^2^, Lei Li ^2^, Lin Wang ^1^, Dao Wen Wang ^1^, Yunzeng Zou ^2^

1 Division of Cardiology, Department of internal medicine, Tongji Hospital, Tongji Medical College, Huazhong University of Science and Technology, Wuhan, China

2 Shanghai Institute of Cardiovascular Diseases, Zhongshan Hospital and Institutes of Biological Science, Fudan University, Shanghai, China

3 Division of Physiology, Department of Basic Sciences, School of Medicine, Loma Linda University, Loma Linda, CA, USA

**Supplementary Methods**

**Human heart samples**

Human failing heart samples were collected from the left ventricles of dilated cardiomyopathy patients undergoing heart transplants. Control samples were obtained from the left ventricles of normal heart donors of traffic accident victims. The samples were obtained after informed consent and with approval of the local Ethical Committee (Tongji Hospital, Tongji Medical College, Huazhong University of Science and Technology, Human Research Ethics Committee, Wuhan, China). The investigation conforms to the principles outlined in the Declaration of Helsinki.

**Isolation and culture of neonatal rat cardiac myocytes**

Primary cultures of neonatal rat cardiac myocytes (NRCMs) were prepared as described previously [[1-3](#_ENREF_1)]. Briefly, 1- to 2-day-old Sprague-Dawley rats were sacrificed by swift decapitation and their hearts were immediately removed under aseptic conditions and placed in ice-cold sterile PBS (calcium- and magnesium-free). Enzymatic and mechanical dissociation of cardiomyocytes was then performed using the Neonatal Cardiomyocyte Isolation System supplied by Worthington Biochemicals (USA). The hearts were minced on ice and digested 15min for 7-8 times with purified trypsin (10 µg/ml) in PBS at 37°C with intermittent gentle swirling. Mild trituration was used to dissociate the digested tissue mechanically, and single-cell suspensions were obtained by filtering this digested material through 70 µm sterile mesh filters. The cells were collected by low-speed centrifugation (1500 rpm for 10 min at room temperature). The supernatant was discarded and the cell pellet resuspended in DMEM (high glucose) culture medium containing 10% FCS (Hyclone Laboratories, USA). Dispersed cells were preplated for 90 min to remove fibroblasts and other proliferation cells, and unattached cells counted and seeded onto 6-well culture plates at a density of 1×10^6^/well. The media was changed every 48 hours beginning the day after seeding. Bromodeoxyuridine (0.1 mM) was added to the culture media for the first 72 h to minimize contamination from fibroblasts. Using this method, we routinely obtained primary cultures with >95% myocytes, as assessed by microscopic observation of spontaneous contraction and by immunocytochemical staining with a monoclonal anti-cardiac α-myosin heavy chain antibody. Cell viability was determined by measuring the cell number, frequency of contractions, cellular morphology, and trypan blue exclusion.

**Treatment of neonatal rat cardiac myocytes**

Neonatal rat cardiomyocytes were cultured in silicon-based plates for 24 hours and then mechanically stretched to 120% followed by treatment with adenovirus as described previously[[3](#_ENREF_3)]. To over-express HSF1 or Smad3, we used replication-defective adenoviral vectors encompassing the entire coding region of the HSF1 or Smad3 gene under the control of the cytomegalovirus promoter. A similar adenoviral vector encoding the green fluorescent protein (GFP) gene was used as a control. To knock down HSF1 expression, shHSF1 was commercially purchased from Santa Cruz Biotechnology, Inc. Next, we generated three AdshHSF1 adenoviruses and selected the one that produced a significant decrease in HSF1 levels for further experiments. AdshRNA was used as the non-targeting control. We infected cells with AdHSF1, AdGFP, AdshHSF1, AdshRNA or AdSmad3 at a MOI of 100, which resulted in transgene expression without toxicity in 95-100% of the cells. Neonatal rat cardiomyocytes were mechanical stretched after infection for respective adenovirus for 24 hours.

**Treatment of the mice with** **isoproterenol**

HSF1 TG, KO mice and their WT littermates (aged 8-10 weeks) were administered with isoproterenol (15mg/kg/d) or 0.9% saline through osmotic minipumps (Model 2004, DURECT, Cupertino, USA) implanted subcutaneously into the back of mice. All animal experimental protocols were approved by the Animal Care and Use Committee of Fudan University and in compliance with “Guide for the Care and Use of Laboratory Animals (the Guide, NRC 2011)”.

**Supplementary Tables**

Supplementary Table 1: A list of primers used in this study.

Genes Forward primer (5′--3`) Reverse primer (5′--3`) GenBank Accession

Human

ANP CAACGCAGACCTGATGGATTT AGCCCCCGCTTCTTCATTC NM_006172

BNP TGGAAACGTCCGGGTTACAG CTGATCCGGTCCATCTTCCT NM_002521

Coll.I GAGGGCCAAGACGAAGACATC CAGATCACGTCATCGCACAAC NM_000088

Coll.III GGAGCTGGCTACTTCTCGC GGGAACATCCTCCTTCAACAG NM_000090

GAPDH TGTGGGCATCAATGGATTTGG ACACCATGTATTCCGGGTCAAT NM_014364

Rat

ANP CCTGGACTGGGGAAGTCAAC ATCTATCGGAGGGGTCCCAG NM_012612.2

BNP TGACGGGCTGAGGTTGTTTT ACACTGTGGCAAGTTTGTGC NM_031545.1

CTGF TGTGAAGACCTACCGGGCTA TTCATGATCTCGCCATCGGG NM_022266.2

GAPDH TCTCTGCTCCTCCCTGTTCT ACCAGCTTCCCATTCTCAGC NM_017008.4

Mouse

ANP GCTTCCAGGCCATATTGGAG GGGGGCATGACCTCATCTT NM_008725

BNP GAGGTCACTCCTATCCTCTGG GCCATTTCCTCCGACTTTTCTC NM_008726

Coll.I GCTCCTCTTAGGGGCCACT CCACGTCTCACCATTGGGG NM_007742

Coll.III CTGTAACATGGAAACTGGGGAAA CCATAGCTGAACTGAAAACCACC NM_009930

GAPDH AATGGATTTGGACGCATTGGT TTTGCACTGGTACGTGTTGAT NM_008085

Supplementary Table 2. The parameters of the heart of C57BL/6 mice after sham operation or TAC

|  | Sham | TAC 2-weeks | TAC 4-weeks |
| --- | --- | --- | --- |
| HR (bpm) | 445 ± 26 | 466 ± 34 | 477± 44 |
| Body weight (g) | 24.8 ± 2.5 | 25.5 ± 2.5 | 26.3 ± 2.4 |
| SBP (mmHg) | 95.6 ± 6.5 | 154.4±8.3^*^ | 150.2±8.8^*^ |
| DBP (mmHg) | 69.9 ± 6.1 | 90.7±7.7^*^ | 87.7±6.9^*^ |
| LVAWd (mm) | 0.75±0.07 | 1.07±0.1^*^ | 0.88±0.09 |
| LVIDd (mm) | 2.13±0.11 | 2.09±0.14 | 2.76±0.2^*#^ |
| EF (%) | 74.3±4.4 | 79.3±4.7 | 50.9±5.1^*#^ |
| LVW/TL (mg/mm) | 3.6±0.3 | 5.6±0.5^*^ | 5.9±0.5^*^ |
| LW/TL (mg/mm) | 7.8±0.7 | 8.1±0.7^*^ | 13.9±1.1^*#^ |
| CSA (μm^2^) | 196±17.3 | 348±24.6^*^ | 336±27.9^*^ |

Values are shown as Means ± SEM. *HR* heart rate, *SBP* end-systolic aortic blood pressure, *DBP* end-diastolic aortic blood pressure, *LV* left ventricular, *LVAWd* LV end-diastolic anterior wall thickness, *LVIDd* LV end-diastolic internal diameter, *EF* LV ejection fraction, *LVW/TL* the ratio of LV weight/tibia length, *LW/TL* the ratio of lung weight/tibia length, *CSA* cross-sectional area of cardiomyocytes. ^*^*P*<0.05 vs sham, ^#^*P*<0.05 vs TAC 2 Weeks. Two-way ANOVA test was used.

Supplementary Table 3: Biometric parameters of 10-week-old WT and HSF1 TG mice at basal condition before operation

|  | WT | HSF1 TG |
| --- | --- | --- |
| HR (bpm) | 445 ± 26 | 461 ± 34 |
| Body weight (g) | 24.8 ± 2.5 | 25.6 ± 3.2 |
| SBP (mmHg) | 95.6 ± 6.5 | 98.5 ± 9.5 |
| DBP (mmHg) | 68.7 ± 5.8 | 68.1 ± 6.8 |
| LVAWd (mm) | 0.75±0.07 | 0.71±0.06 |
| LVIDd (mm) | 2.13±0.11 | 2.11±0.17 |
| EF (%) | 74.3±4.4 | 71.3±4.9 |
| LVW/TL (mg/mm) | 3.6±0.3 | 3.7±0.4 |
| LW/TL (mg/mm) | 7.8±0.7 | 7.5±0.4 |
| CSA (μm^2^) | 196±17.3 | 211±19.6 |

Values represent mean ± SEM. *HR* heart rate, *SBP* end-systolic aortic blood pressure, *DBP* end-diastolic aortic blood pressure, *LV* left ventricular, *LVAWd* LV end-diastolic anterior wall thickness, *LVIDd* LV end-diastolic internal diameter, *EF* LV ejection fraction, *LVW/TL* the ratio of LV weight/tibal length, *LW/TL* the ratio of lung weight/tibal length, *CSA* cross-sectional area of cardiomyocytes. Two-way ANOVA test was used.

Supplementary Table 4. The parameters of the heart of HSF1 TG mice and their littermates after TAC or sham operation.

|  | | WT | | HSF1 TG | |
| --- | --- | --- | --- | --- | --- |
|  | Sham | TAC-4weeks | Sham | | TAC-4weeks |
| HR (bpm) | 464 ± 31 | 484± 44 | 458 ± 22 | | 483± 44 |
| Body weight (g) | 24.3 ± 2.1 | 26.7 ± 2.6 | 24.9 ± 2.1 | | 27.2 ± 2.2 |
| SBP (mmHg) | 99.1 ± 6.5 | 155.2±7.6^*^ | 97.6 ± 6.5 | | 158.3±11.5^*^ |
| DBP (mmHg) | 70.6 ± 6.9 | 90.3±8.9^*^ | 71.2 ± 6.9 | | 93.9±7.1^*^ |

Values are shown as mean ± SEM. *HR* heart rate, *SBP* end-systolic aortic blood pressure, *DBP* end-diastolic aortic blood pressure; ^*^*P*<0.05 vs respective sham. Two-way ANOVA test was used.

Supplementary Table 5: Biometric parameters of 10 weeks old WT and HSF1 KO mice at basal condition before operation

|  | WT | HSF1 KO |
| --- | --- | --- |
| HR (bpm) | 441 ± 56 | 456± 51 |
| Body weight (g) | 25.1 ± 3.1 | 25.0 ± 2.4 |
| SBP (mmHg) | 98.6 ± 6.9 | 97.2±6.8 |
| DBP (mmHg) | 66.9 ± 7.1 | 67.7±6.9 |
| LVAWd (mm) | 0.79±0.09 | 0.80±0.09 |
| LVIDd (mm) | 2.16±0.14 | 2.16±0.2 |
| EF (%) | 73.7±4.8 | 74.9±5.1 |
| LVW/TL (mg/mm) | 3.5±0.5 | 3.9±0.6 |
| LW/TL (mg/mm) | 7.6±0.7 | 7.9±0.9 |
| CSA (μm^2^) | 203±16.1 | 206±20.9 |

Values are shown as Mean ± SEM. *HR* heart rate, *SBP* end-systolic aortic blood pressure, *DBP*end-diastolic aortic blood pressure, *LV* left ventricular, *LVAWd* LV end-diastolic anterior wall thickness, *LVIDd* LV end-diastolic internal diameter, *EF* LV ejection fraction, *LVW/TL* the ratio of LV weight/tibal length, *LW/TL* the ratio of lung weight/Tibal length, *CSA* cross-sectional area of cardiomyocytes. Two-way ANOVA test was used.

Supplementary Table 6. The parameters of the heart of HSF1 KO mice and their littermates after TAC or sham operation.

|  | WT | | HSF1 KO | |
| --- | --- | --- | --- | --- |
|  | Sham | TAC-4weeks | Sham | TAC-4weeks |
| HR (bpm) | 459 ± 31 | 477± 38 | 468 ± 22 | 492± 49 |
| Body weight (g) | 24.9 ± 2.1 | 27.4 ± 2.6 | 25.4 ± 2.5 | 27.9 ± 2.9 |
| SBP (mmHg) | 97.8 ± 8.5 | 157.1 ± 9.9^*^ | 99.7 ± 7.3 | 152.4 ± 12.5^*^ |
| DBP (mmHg) | 69.6 ± 7.7 | 90.3 ± 8.9^*^ | 71.2 ± 6.9 | 93.9 ± 7.1^*^ |

Values are shown as mean ± SEM. *HR* heart rate, *SBP* end-systolic aortic blood pressure, *DBP* end-diastolic aortic blood pressure. ^*^*P*<0.05 vs respective sham. Two-way ANOVA test was used.

Supplementary table 7. The parameters of the heart of HSF1 TG mice and their littermates after treatment with vehicle or ISO for 4 weeks

|  | WT | | HSF1 TG | |
| --- | --- | --- | --- | --- |
|  | Vehicle | ISO | Vehicle | ISO |
| HR (bpm) | 486 ± 42 | 499± 58 | 480 ± 52 | 468± 59 |
| Body weight (g) | 25.9 ± 2.3 | 24.4 ± 3.6 | 26.1 ± 2.5 | 25.9 ± 3.2 |
| SBP (mmHg) | 93.7 ± 8.1 | 144.1 ± 11.9^*^ | 96.7 ± 8.4 | 146.3 ± 10.5^*^ |
| DBP (mmHg) | 65.9 ± 7.6 | 97.3 ± 7.9^*^ | 70.2 ± 6.9 | 95.9 ± 7.6^*^ |
| LVAWd (mm) | 0.75±0.09 | 0.84±0.08 | 0.77±0.06 | 0.75±0.06 |
| LVIDd (mm) | 2.12±0.18 | 4.09±0.2^*^ | 2.21±0.19 | 2.14±0.2 |
| EF (%) | 73.8±5.9 | 48.6±5.7^*^ | 75.1±4.8 | 70.9±6.1^#^ |
| LVW/TL (mg/mm) | 3.7±0.3 | 5.8±0.5^*^ | 3.8±0.5 | 4.2±0.6^#^ |
| LW/TL (mg/mm) | 7.6±0.5 | 13.8±1.5^*^ | 7.6±0.9 | 8.4±0.7^#^ |
| CSA (μm^2^) | 212±19.1 | 339±19.9^*^ | 203±18.1 | 223±27.9^#^ |
| MFA (%) | 1.4±0.12 | 4.7±0.25^*^ | 1.2±0.15 | 1.6±0.16^#^ |
| PVF (%) | 10.7±1.1 | 54.5±4.5^*^ | 10.8±1.2 | 15.9±2.6^#^ |
| Coll . I | 1.0±0.11 | 4.7±0.35^*^ | 1.12±0.12 | 1.7±0.12^#^ |
| Coll.III | 1.0±0.12 | 5.55±0.25^*^ | 1.18±0.2 | 1.82±0.16^#^ |

Values are shown as Mean ± SEM. *HR* heart rate, *SBP* end-systolic aortic blood pressure, *DBP*, end-diastolic aortic blood pressure, *LV* left ventricular, *LVAWd* LV end-diastolic anterior wall thickness, *LVIDd* LV end-diastolic internal diameter, *EF* LV ejection fraction, *LVW/TL* the ratio of LV weight/Tibal length, *LW/TL* the ratio of lung weight/Tibal length, *CSA* cross-sectional area of cardiomyocytes, *MFA* myocardial fibrosis area, *PVF* perivascular fibrosis area. Coll. I and Coll. III are relative mRNA level compared to WT-Vehicle group. ^*^*P*<0.01 vs respective vehicle, ^#^*P*<0.01 vs WT-ISO. Two-way ANOVA test was used.

.

Supplementary Table 8. The parameters of the heart of HSF1 KO mice and their littermates after treatment with vehicle or ISO for 4 weeks

|  | WT | | HSF1 KO | |
| --- | --- | --- | --- | --- |
|  | Vehicle | ISO | Vehicle | ISO |
| HR (bpm) | 477 ± 48 | 489± 52 | 468 ± 62 | 478± 49 |
| Body weight (g) | 25.2 ± 2.6 | 23.4 ± 2.6 | 25.6 ± 2.5 | 24.1 ± 3.2 |
| SBP (mmHg) | 93.1 ± 6.7 | 149.2 ± 12.4^*^ | 92.1 ± 5.4 | 146.5 ± 12.5^*^ |
| DBP (mmHg) | 65.1 ± 8.6 | 93.6 ± 6.7^*^ | 66.9 ± 5.9 | 92.8 ± 6.6^*^ |
| LVAWd (mm) | 0.72±0.05 | 0.79±0.05 | 0.77±0.08 | 0.73±0.09 |
| LVIDd (mm) | 2.19±0.13 | 4.11±0.4^*^ | 2.27±0.21 | 4.91±0.2^*#^ |
| EF (%) | 70.4±7.1 | 48.2±5.3^*^ | 72.7±5.2 | 39.4±3.1^*#^ |
| LVW/TL (mg/mm) | 3.8±0.2 | 5.7±0.3^*^ | 3.4±0.2 | 6.8±0.3^*##^ |
| LW/TL (mg/mm) | 6.6±0.3 | 12.4±1.5^*^ | 6.9±0.3 | 18.4±0.7^*##^ |
| CSA (μm^2^) | 221±16.7 | 338±11.9^*^ | 213±17.3 | 363±27.9^*##^ |
| MFA (%) | 1.4±0.12 | 4.6±0.3^*^ | 1.2±0.15 | 8.7±0.16^*##^ |
| PVF (%) | 9.8±0.8 | 55.1±5.5^*^ | 10.7±1.1 | 78.1±4.6^*##^ |
| Coll . I | 1.0±0.12 | 4.2±0.15^*^ | 1.11±0.12 | 6.9±0.12^*##^ |
| Coll.III | 1.0±0.13 | 5.7±0.3^*^ | 1.2±0.2 | 9.2±0.16^*##^ |

Values are shown as Mean ± SEM. *HR* heart rate，*SBP* end-systolic aortic blood pressure，*DBP* end-diastolic aortic blood pressure，*LV* left ventricle, *LVAWd* LV end-diastolic anterior wall thickness, *LVIDd* LV end-diastolic internal diameter, *EF* LV ejection fraction, *LVW/TL* the ratio of LV weight/tibal length, *LW/TL* the ratio of lung weight/tibal length, *CSA* cross sectional area of cardiomyocytes, *MFA*, myocardial fibrosis area, *PVF* perivascular fibrosis area. Coll. I and Coll. III are relative mRNA levels compared to WT-Vehicle group. ^*^*P*<0.01 vs respective vehicle, ^#^*P*<0.05, ^##^*P*<0.01 vs WT-ISO. Two-way ANOVA test was used.

**Supplementary Figures**

**Donor hearts**

**DCM hearts**

**relative mRNA**

***Coll. I***

***Coll. III***

**20**

**10**

**0**

*****

*****

**5**

**15**

**25**

**relative mRNA**

***ANP***

***BNP***

**40**

**30**

**20**

**10**

**0**

*****

*****

**Coll. I**


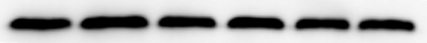

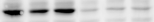

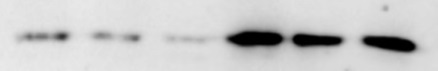


**Coll. III**

**GAPDH**

**Donor hearts**

**DCM hearts**

**B**

**C**

**A**

**25**

**20**

*****

**relative protein**

**10**

*****

**15**

**Coll. I**

**Coll. III**

**0**

**5**

**relative *p*-HSF1/t-HSF1 ratio**

**0.8**

**0.2**

**0**

**1.2**

*****

**0.4**

**0.6**

**1.0**

***t*-HSF1**


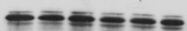


***p*-HSF1**


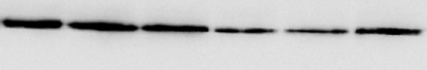


**Donor hearts**

**DCM hearts**

**GAPDH**


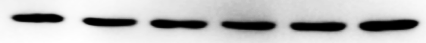

**1**

**0**

**3**

**2**

**D**

*****


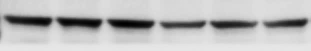


***p*-Smad3**


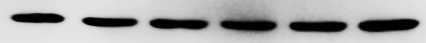


***p*-Smad3/total Smad3 ratio**

***t*-Smad3**


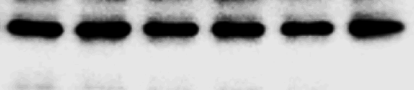


**GAPDH**

**Donor hearts**

**DCM hearts**

Supplementary Fig.1 HSF1 phosphorylation was decreased in human failing hearts. (A) Real-time PCR analysis of ANP, BNP, Coll. I and Coll.III in human failing hearts and donor hearts. B, C and D, Representative western blots of Coll. I, Coll. III, total HSF1 (*t*-HSF1) and phosphorylated HSF1 (*p*-HSF1), total Smad3 (*t*-Smad3) and phosphorylated Smad3 (*p*-Smad3) in human failing hearts and donor hearts (n=6). Values represent mean ± SEM. ^*^*P*<0.01 for failing hearts vs. donor hearts. Two-way ANOVA test was used.


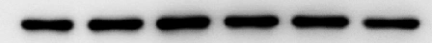

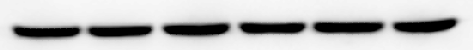


**WT**

**HSF1 TG**


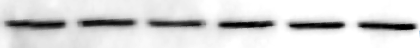

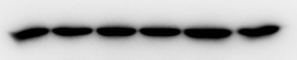


***t*-HSF1**

***p*-HSF1**

**GAPDH**


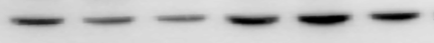

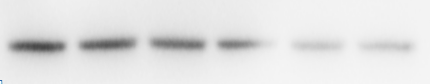


**WT**

**HSF1 KO**

***p*-HSF1/total HSF1**

**(relative folds)**

**0.8**

**0.4**

**0**

**1.2**

*****

**WT**

**HSF1 KO**

**1.0**

**0.6**

**0.2**

***p*-HSF1/total HSF1**

**(relative folds)**

**2**

**1**

**0**

**4**

*****

**WT**

**HSF1 TG**

**3**

Supplementary Fig.2 The expression and phosphorylation of HSF1 in HSF1 TG and KO mouse hearts. Representative western blots of total HSF1 (*t*-HSF1) and phosphorylated HSF1 (*p*-HSF1) in indicated mouse hearts (n=5). Below are quantitative results. Values represent mean ± SEM. ^*^*P*<0.01 for failing hearts vs. respective WT littermates. Two-way ANOVA test was used.

**control**

**stretch**

A

*****

**#**

**6**

**4**

**5**

*****

*****

**relative *CTGF* mRNA level**

**3**

**2**

**#**

**1**

**0**

**#**

AdGFP

AdHSF1

AdShRNA

AdShHSF1

**6**

*****

**7**

B

**5**


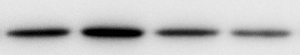

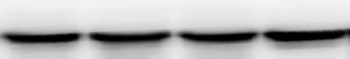

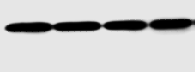

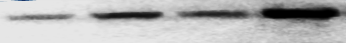


CTGF

GAPDH

Stretch

-

**+**

-

**+**

-

**+**

-

**+**

AdGFP

AdHSF1

AdshRNA

AdshHSF1

**4**

**relative *CTGF* protein level**

**3**

*****

*****

**2**

**1**

**#**

**0**

AdGFP

AdHSF1

AdShRNA

AdShHSF1

Supplementary Fig.3 HSF1 inhibits mechanical stretch induced-fibrotic response in cardiomyocytes (CMs) upon mechanical stretch. (A) Real-time polymerase chain reaction (PCR) analysis of connective tissue growth factor (CTGF) in CMs infected with indicated adenoviruses after the treatment with mechanical stretch for 24 hours (n=4 independent experiments). (B) The protein levels of CTGF in CMs. Left, representative western blots of CTGF in indicated CMs (n=4 independent experiments). Right, quantitative results. Values represent mean ± SEM. ^*^*P*<0.01 for stretched CMs vs. respective control. Two-way ANOVA test was used.

TAC

sham

IP: Smad3

WT

KO

TG

WB: HSP70

WBP: HSP90


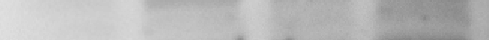

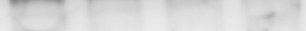

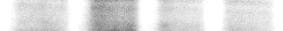

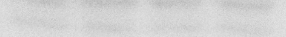

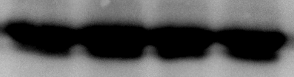

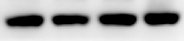


WT

TAC

sham

TAC

sham

TAC

sham

Supplementary Fig.4 No bindings were detected between HSP70/HSP90 and Smad3. The left ventricular tissue extracts were used to do immunoprecipitation. No HSP70/HSP90 was detected in the immunoprecipitation complex. Representative western blots of HSP70 and HSP90 of the above 2 blots. Bottom are WB of total Smad3 in the immunoprecipitation complex (n=4 independent experiments). Two-way ANOVA test was used.

1. Zou Y, Liang Y, Gong H, Zhou N, Ma H, Guan A, Sun A, Wang P, Niu Y, Jiang H, Takano H, Toko H, Yao A, Takeshima H, Akazawa H, Shiojima I, Wang Y, Komuro I, Ge J (2011) Ryanodine Receptor Type 2 Is Required for the Development of Pressure Overload-Induced Cardiac Hypertrophy. Hypertension 58: 1099-U1363. DOI 10.1161/hypertensionaha.111.173500

2. Zou Y, Li J, Ma H, Jiang H, Yuan J, Gong H, Liang Y, Guan A, Wu J, Li L, Zhou N, Niu Y, Sun A, Nakai A, Wang P, Takano H, Komuro I, Ge J (2011) Heat shock transcription factor 1 protects heart after pressure overload through promoting myocardial angiogenesis in male mice. Journal of Molecular and Cellular Cardiology 51: 821-829. DOI 10.1016/j.yjmcc.2011.07.030

3. Zhou N, Li L, Wu J, Gong H, Niu Y, Sun A, Ge J, Zou Y (2010) Mechanical stress-evoked but angiotensin II-independent activation of angiotensin II type 1 receptor induces cardiac hypertrophy through calcineurin pathway. Biochemical and Biophysical Research Communications 397: 263-269. DOI 10.1016/j.bbrc.2010.05.097
